# Diagnosis of Parkinson's Disease Based on Disease-Specific Autoantibody Profiles in Human Sera

**DOI:** 10.1371/journal.pone.0032383

**Published:** 2012-02-22

**Authors:** Min Han, Eric Nagele, Cassandra DeMarshall, Nimish Acharya, Robert Nagele

**Affiliations:** 1 University of Medicine and Dentistry of New Jersey-Graduate School of Biomedical Sciences at the School of Osteopathic Medicine, Stratford, New Jersey, United States of America; 2 New Jersey Institute for Successful Aging, University of Medicine and Dentistry of New Jersey, Stratford, New Jersey, United States of America; 3 Durin Technologies, Inc. New Brunswick, New Jersey, United States of America; Johns Hopkins, United States of America

## Abstract

Parkinson's disease (PD), hallmarked by a variety of motor disorders and neurological decline, is the second most common neurodegenerative disease worldwide. Currently, no diagnostic test exists to identify sufferers, and physicians must rely on a combination of subjective physical and neurological assessments to make a diagnosis. The discovery of definitive blood-borne biomarkers would be a major step towards early and reliable diagnosis. Despite attention devoted to this search, such biomarkers have remained elusive. In the present study, we used human protein microarrays to reveal serum autoantibodies that are differentially expressed among PD and control subjects. The diagnostic significance of each of these autoantibodies was evaluated, resulting in the selection of 10 autoantibody biomarkers that can effectively differentiate PD sera from control sera with a sensitivity of 93.1% and specificity of 100%. PD sera were also distinguishable from sera obtained from Alzheimer's disease, breast cancer, and multiple sclerosis patients with accuracies of 86.0%, 96.6%, and 100%, respectively. Results demonstrate that serum autoantibodies can be used as highly specific and accurate biomarkers for PD diagnosis throughout the course of the disease.

## Introduction

Parkinson's disease (PD) is a chronic and progressive motor system disorder inflicting profound social and economic costs worldwide. It is the second most common neurodegenerative disorder after Alzheimer's disease (AD), affecting more than 1% of 55-year-old individuals and more than 3% of those over the age of 75 [1]. The primary symptoms of PD include tremor, rigidity, bradykinesia, and postural instability [2]. The cardinal pathological feature of PD is the loss of dopaminergic neurons in the substantia nigra, a brain region involved in coordination and control of muscle activity [3], [4]. Although PD manifests primarily as a motor disability, recent studies reveal many pre-motor symptoms that suggest an onset of PD pathology years before characteristic symptoms appear [5]–[7]. By the time a diagnosis is made, at least one-third of substantia nigra neurons and striatal dopaminergic fibers are already lost [8], [9].

Despite years of research, there is no one test or technique that can provide a conclusive primary diagnosis of PD. Current diagnostic methods are based on medical history evaluation and a combination of physical and neurological assessments [10], [11]. Standard practices for these assessments, such as the Unified Parkinson's Disease Rating Scale (UPDRS) [12], [13], have aided tremendously in clinical staging of the disease, but fail to detect PD before the onset of initial motor symptoms. Additional techniques, such as CT, MRI, and PET neuroimaging, may be used to rule out other neurological disorders, but rarely do they detect any abnormality that can be directly related to the onset of PD [14]. There are also no laboratory tests utilizing blood, cerebrospinal fluid, or urine samples that have proven to be effective in primary diagnosis or confirmation of PD. Thus, there is still pressing need for an accurate, relatively non-invasive, and affordable PD diagnostic test. This is particularly true given widespread recognition that early detection facilitating early treatment helps to slow the progression of the disease, minimize symptoms, and improve the overall quality of life [15].

We have recently demonstrated the ubiquitous presence of autoantibodies in human sera, regardless of patient age or health status [16], [17]. These findings led us to test the hypothesis that the presence of ongoing disease causes consistent, disease-specific perturbations of autoantibody profiles in the blood. In the case of AD, we have previously used human protein microarrays to compare disease and control serum autoantibody profiles and detected disease-specific autoantibody biomarkers capable of differentiating blinded AD and control serum samples with a sensitivity of 96.0% and specificity of 92.5% [18]. In the present study, we again used human protein microarrays to detect and measure disease group- and control group-specific variations in autoantibody expression patterns in an effort to identify potential diagnostic biomarkers of PD. Our results confirm that autoantibody expression profiles can be used to select a relatively small subset of autoantibody biomarkers that can detect the presence of PD with great accuracy and specificity using only a small sample of blood.

## Materials and Methods

### Ethics Statement

Approval for the use of blood samples for this study was obtained from the UMDNJ-Stratford Institutional Review Board.

### Human Serum Samples

Twenty-nine Parkinson's disease (PD) serum samples, 50 AD samples, and 40 control samples were obtained from *Analytical Biological Systems, Inc.* (Wilmington, DE). Thirty breast cancer (BC) serum samples and 10 multiple sclerosis (MS) serum samples were obtained from *Asterand, Inc.* (Detroit, MI). In an attempt to develop a diagnostic with broad application to all PD patients at all stages of the disease, our PD serum pool contained samples from early, progressive and late stage PD subjects. All samples were handled by standard procedures and stored at −80°C. Diagnosis of PD was based on a clinical evaluation based on Gelb criteria [11]. Demographic characteristics of the study population are shown in Table 1.

**Table 1:** Demographics of Serum Donors.

Group	n	Age		Sex
		Mean	Range	(% male)
**Parkinson's Disease**	29	74.0	53–88	55%
**Alzheimer's Disease**	50	78.5	61–97	40%
**Multiple Sclerosis**	10	46.0	27–59	30%
**Breast Cancer**	30	46.7	32–54	0%
**Controls**	40	40.4	19–86	82%
**–Older Control**	20	57.7	51–86	100%
**–Younger Control**	20	24.7	19–30	65%

### Human Protein Microarrays

To identify autoantibodies in human sera, we used *Invitrogen's* ProtoArray v5.0 Human Protein Microarrays (Cat. *No.* PAH0525020, *Invitrogen*, Carlsbad, CA, USA), each containing 9,486 unique human protein antigens (www.invitrogen.com/protoarray). All proteins were expressed as GST fusion proteins in insect cells, purified under native conditions, and spotted in duplicate onto nitrocellulose-coated glass slides. All arrays were probed and scanned according to the manufacturer's instructions using commercially prepared reagents. Briefly, microarray slides were blocked (Blocking Buffer, Cat. *No. PA055*, *Invitrogen*) and then incubated with serum samples, diluted 1∶500 in washing buffer. After washing, the arrays were probed with anti-human IgG (H+L) conjugated to AlexaFluor 647 (Cat. *No.* A-21445, Invitrogen). Arrays were then washed, dried, and immediately scanned with a GenePix 4000B Fluorescence Scanner (*Molecular Devices*, Sunnyvale, CA, USA).

### Microarray Data Analysis

The fluorescence data from each microarray was acquired by *Genepix Pro* analysis software after scanning, and then synced with Invitrogen's lot-specific *Genepix Array List* (GAL) files. The resulting *Genepix Results* (GPR) files were then imported into Invitrogen's *Prospector 5.2* for analysis. All data is MIAME compliant and the raw data has been deposited in a MIAME compliant database (GEO) under the accession number GSE29654. The “group characterization” and “two - group comparison” features in the *IRBP Toolbox* allowed for M-statistical analysis of autoantibody expression. Sorting detectable autoantibodies by difference of prevalence between PD and control groups in descending order, we selected the top 10 as our potential diagnostic biomarkers.

The selected biomarkers were re-verified as significant by *Predictive Analysis for Microarrays* (*PAM*) – an independent algorithm relying on nearest shrunken centroid analysis to identify proteins acting as significant class-differentiators. The predictive classification accuracy of the identified biomarkers was tested with *Random Forest* (*RF*) using the default settings, another significance algorithm run as an *R* package (v 2.12.1). In *RF*, partitioning trees are built by successively splitting the samples according to a measure of statistical impurity at a given node until terminal nodes are as homogenous as possible. Classification accuracy for a given set of diagnostic biomarkers is reported in a confusion matrix and misclassification as an Out-Of-Bag (OOB) error score.

## Results

### Selection of Autoantibody Biomarkers for PD Diagnosis

**Figure 1:**
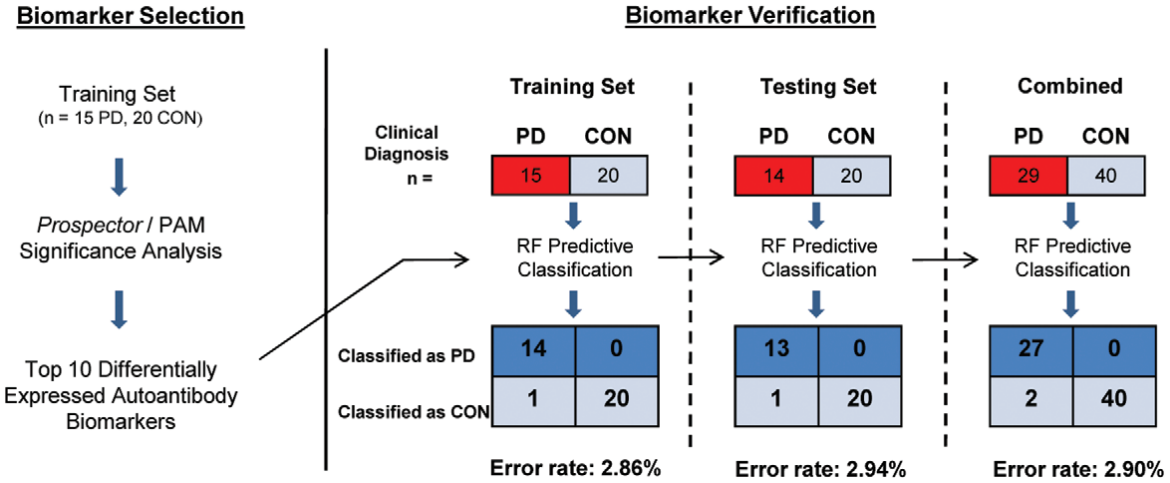
Biomarker selection and training/testing analysis. Before biomarker selection, our total sample pool was split into two randomized groups: the Training Set and Testing Set. *Prospector* and *PAM* statistical analyses were performed on the Training Set to identify the top 10 most significant autoantibody classifiers of PD and control. We then verified the diagnostic accuracy of these selected biomarkers by using *Random Forest* to predict sample classification in the Training Set, Testing Set, and then both sets combined.

**Figure 2:**
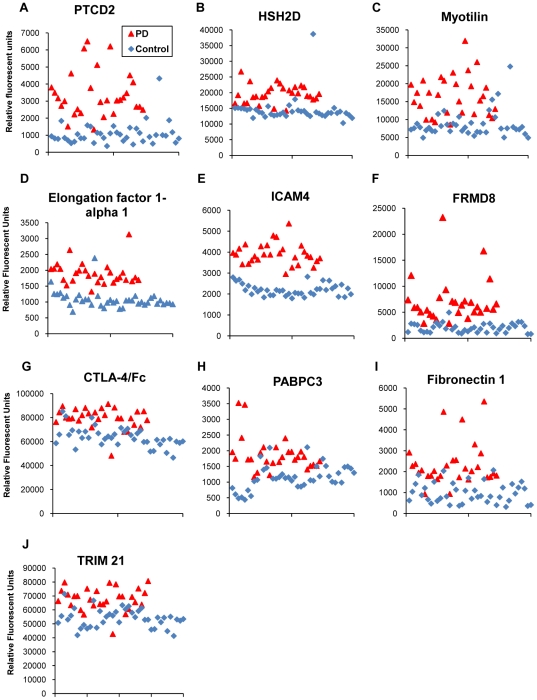
Differential expression of identified PD-specific autoantibody biomarkers in PD and control sera. Microarray fluorescence values reflecting individual serum autoantibody titers demonstrate the differences in the serum expression of the selected ten PD-specific autoantibody biomarkers in PD (n = 29) and control (n = 40) sera (A–I).

A total of 69 human serum samples (29 PD and 40 controls; Table 1) were assigned to either a Training Set (15 PD, 20 control) or Testing Set (14 PD, 20 control), each containing equal proportions of early-, progressive-, and late-stage PD samples as well as older and younger controls. To identify potential diagnostic autoantibodies for PD, we probed human protein microarrays, each containing 9,486 native antigens, with Training Set sera and analyzed the data as described in Materials and Methods (Fig. 1). *Prospector* analysis software determined that 780 autoantibodies had a significantly higher prevalence in the PD group than in the control group (p<0.01) and thus represent potential PD biomarkers. We selected the 10 autoantibody biomarkers that demonstrated the largest difference in group prevalence between PD and controls to serve as our diagnostic indicators (Table 2). The differential expression of these 10 autoantibody biomarkers is shown in Figure 2. As an independent verification of the 10 biomarkers selected, we re-evaluated our data with *Predictive Analysis for Microarrays* (*PAM*) [19]. *PAM* confirmed that the 10 biomarkers originally selected by *Prospector* were among the most significant classifiers of PD and controls.

**Table 2:** Identity and Significance of 10 PD vs. Control Diagnostic Biomarkers.

Database ID	Description	Prevalence in PD	Prevalence in Controls	*p*
**NM_001544.2**	Intercellular adhesion molecule 4 (Landsteiner-Wiener blood group) (ICAM4), transcript variant 1	93.55%	2.38%	1.73E-18
**NM_024754.2**	Pentatricopeptide repeat domain 2 (PTCD2)	90.32%	7.14%	9.40E-13
**BC051695.1**	FERM domain containing 8 (FRMD8)	87.10%	4.76%	1.31E-14
**PHR5001**	Recombinant human CTLA-4/Fc	87.10%	14.29%	6.14E-11
**NM_006790.1**	Myotilin (MYOT)	90.32%	21.43%	5.66E-10
**NM_032855.1**	Hematopoietic SH2 domain containing (HSH2D)	87.10%	7.14%	1.71E-13
**BC005858.1**	Fibronectin 1 (FN1)	90.32%	14.29%	7.39E-08
**NM_003141.2**	Tripartite motif-containing 21 (TRIM21)	80.65%	9.52%	1.07E-10
**BC094687.1**	Elongation factor 1-alpha 1	87.10%	7.14%	3.03E-10
**BC027617.1**	Poly(A) binding protein, cytoplasmic 3 (PABPC3)	74.19%	11.91%	0.000805

### Verification of Biomarkers via Training and Testing Set Analysis

To assess the Training and Testing set classification accuracies of the 10 selected PD biomarkers, we used *Random Forest* (*RF*) [20]. *RF* is a statistical algorithm that creates voting classes of decision-making trees to evaluate the significance of each marker and classify samples. Using our 10 biomarkers to “diagnose” the Training Set (n = 35; 15 PD and 20 control), *RF* had an overall accuracy of 97.1% [Out-of-Bag (OOB) Error 2.9%, a positive predictive value (PPV) of 100%, and a negative predictive value (NPV) of 95.2%]. When the same 10 biomarkers were used to classify Testing Set sera (n = 34; 14 PD and 20 control), which played no part in the biomarker selection process, *RF* distinguished PD samples from controls with equal accuracy (prediction error of 2.9%, PPV of 100.0%, and NPV of 95.2%). When the 10 autoantibody biomarkers were used to classify all PD and control samples simultaneously (n = 69; 29 PD, 40 control) in *RF*, they did so with a 93.1% sensitivity and 100% specificity.

### Differentiation of PD from Other Diseases

Using the 10 selected autoantibody biomarkers, PD samples were correctly differentiated from controls with a high and consistent accuracy (Table 3). But to test the biomarkers for disease specificity, we sought to differentiate PD from other non-neurological and neurological diseases. To accomplish this, we used our 10 selected biomarkers to differentiate 30 breast cancer serum samples from the 29 PD samples. *RF* reported an OOB Error of 3.39% (PPV and NPV of 93.5% and 100%, respectively). These results are similar to those of the PD versus control trials described above and demonstrate that there is no diagnostic bias toward disease in general. To verify biomarker specificity against another central nervous system disorder, we used Multiple Sclerosis (MS) sera as a neurologically diseased control. Results show that our 10 PD autoantibody biomarkers can distinguish PD and MS samples with 100% accuracy (Table 3).

**Table 3:** Diagnostic Accuracies of Selected Biomarkers.

	PD (n = 29) vs.					
	All Controls	Older Control	Younger Control	AD*	Breast Cancer	MS
	n = 40	n = 20	n = 20	n = 50	n = 30	n = 10
**Sensitivity %**	93.1	96.6	96.6	79.3	100.0	100.0
**Specificity %**	100.0	100.0	100.0	90.0	93.3	100.0
**PPV%**	100.0	100.0	100.0	82.1	93.5	100.0
**NPV %**	95.2	95.2	95.2	88.2	100.0	100.0

* *The biomarkers used for this classification are those of Table 5 in our previous work* [*18*] *; all others are the biomarkers identified in* *Table 2*.

These results, combined with the previous work in which we demonstrated that PD can be distinguished from AD using only five autoantibody biomarkers [18], provide further confirmation that these biomarkers can be used to generate a specific and reliable PD diagnostic.

## Discussion

Parkinson's disease (PD) is a progressive motor system disorder that affects over five million people worldwide. No diagnostic test is yet available. In addition to causing patient anguish, this lack of confirmation hinders our ability to test potential disease-modifying drugs and other neuroprotective strategies. Identification of early-stage PD is the most difficult to achieve; pre-clinical detection is currently impossible. Identification of blood-borne biomarkers for accurate diagnosis and early detection of PD has long been a major goal since this is required for early patient access to therapy. In the present study, we have confirmed that autoantibody expression profiles can be used to select a relatively small subset of autoantibody biomarkers that can detect the presence of PD with great sensitivity and specificity using only a small sample of blood.

### A PD Diagnostic Based on Disease-Associated Autoantibody Profiles

Using human protein microarrays, we have previously demonstrated that the number of autoantibodies detectable in human sera is surprisingly high, averaging over one thousand as detected by this method but displaying wide individual variation [18]. Although the function of such a large number of autoantibodies is unknown, we have found that the presence of disease causes specific perturbations in autoantibody profiles that are useful for disease detection and diagnosis. It was this differential expression of autoantibodies that allowed us to identify and test diagnostic biomarkers for Alzheimer's disease (AD) [18]. The present study demonstrates that PD is also linked to characteristic alterations in serum autoantibody expression profiles. Just as in AD, these changes allow for the unbiased identification and selection of specific autoantibodies that can effectively function as diagnostic biomarkers. We have shown here that with only 10 autoantibody biomarkers, PD serum samples were readily distinguished from control sera with a sensitivity of 93.1% and a specificity of 100%.

The most rigorous test of the significance and predictive value of diagnostic biomarkers is validation in a variety of circumstances. The 10 PD autoantibody biomarkers were selected using a Training Set of samples and verified using an independent Testing Set of samples that played no role in their selection and still provided a sensitivity of 92.8% and specificity of 100%. Furthermore, their specificity was confirmed by successfully differentiating PD sera from other diseased sera, including MS and breast cancer. Additionally, PD and Alzheimer's disease (AD) are known to be even more closely related and are often co-morbid with many similarities that can sometimes make it difficult to clearly distinguish these two diseases by conventional means alone [21], [22]. We previously demonstrated that with as few as only five autoantibody biomarkers, it was possible to differentiate PD samples from AD samples [18]. Diagnosing PD from AD, breast cancer, and multiple sclerosis, we achieved accuracies of 86.0%, 96.6% and 100%, respectively, demonstrating no diagnostic bias toward disease.

### Multiplicity of Differential Autoantibodies

As in our study of AD biomarkers [18], we detected a large number of differentially expressed autoantibodies in the PD and control groups. *Prospector* identified 96 differentiating autoantibodies with a p-value of less than 0.0001 and group prevalence differences of over 40%, all of which are potentially useful for PD diagnostics. Importantly, this evaluation of significance was duplicated by the other statistical algorithms used here, *PAM* and *RF*. Most autoantibodies considered as significant diagnostic biomarkers by one program were repeatedly selected as significant by the other two. As shown for AD, this finding suggests that many combinations of autoantibody biomarkers can be successfully used to distinguish PD sera from control sera with varying accuracies. Given the large number of differentially expressed autoantibodies present in sera from patients with PD and AD, two high-prevalence neurodegenerative diseases with some common pathology, we find it likely that they share a similar mechanism for autoantibody generation.

### Possible Origin of Diagnostic Autoantibodies

The underlying reason for the presence and abundance of autoantibodies in human sera, especially in younger and healthy individuals, is unknown. Although some autoantibodies may be remnants of past disease and reflect a history of immunological activity, many may also be present as a result of ongoing or current disease. We suggest that the presence of an active disease, resulting in chronic cell damage and death, causes the production and release of cellular debris, some of which is antigenic. For example, in PD, the early and somewhat selective loss of dopaminergic neurons in the substantia nigra would provide a chronic, yet cell-type-specific source of such proteins and their breakdown products. These materials released to the surrounding interstitial fluid would eventually re-enter the blood and lymph, encounter the immune surveillance system and presumably elicit an autoimmune response. We propose that this immune response leads to the production and appearance of a relatively large number of autoantibodies in the blood which could conceivably be involved in clearance of debris generated by the presence of disease. Since different cell types share many common proteins, only a very small subset of protein targets and their corresponding autoantibodies would be expected to be truly cell-type specific, and thus useful for disease detection and diagnostics. A similar scenario has recently been proposed to account for the presence of autoantibodies that useful for the diagnosis of Alzheimer's disease in the blood of patients suffering from this devastating disease [18].

In PD, AD, and a number of other neurodegenerative diseases, earlier stages of disease are known to be more focal and associated with a more selective targeting of specific neuronal subtypes. This selective cell degeneration and death would be expected to initially favor the appearance of a narrower spectrum of autoantibodies that are disease-specific. However, as disease pathology advances in the brain along with widespread inflammation, the declining local conditions would be expected to negatively affect other nearby cell types, thus resulting in their loss and the later appearance of additional autoantibodies reflecting the involvement of these new cell types. In addition, it is well-known that neuronal degeneration in one brain region can induce a subsequent neuronal degeneration in other remote brain regions as a result of lost connectivity, and that these changes can eventually compromise the structural and functional integrity of components of the peripheral nervous system. Thus, the spread of pathology that is common to many neurodegenerative diseases raises the possibility that different disease stages may be distinguishable from one another based on their unique autoantibody profiles that are dictated by their current pathology. Further work will be necessary to test this possibility.

### Benefits of Antigen Identification

One obvious advantage of using protein microarrays to detect disease-associated autoantibodies in sera is that the identities of both the autoantibodies and their antigen targets become known. This knowledge may prove to be beneficial to drug discovery and other therapeutic efforts, especially if these identities shed new light on key components of disease-relevant pathways that can be specifically targeted. Currently, little is known about many of the antigens identified here as targets of PD-specific autoantibody biomarkers. However, some common patterns are beginning to emerge. For example, the biomarker antigen discussed above, FRMD8, has also been shown to be an effective diagnostic indicator for Alzheimer's disease [18]. Overlap of useful diagnostic indicators is not surprising, since both diseases involve the degeneration and death of closely similar cell type (both are brain neurons). As more is learned about the functions of serum autoantibodies and their targets, we anticipate that a better understanding of autoantibody profiles will eventually yield significant research and therapeutic benefits.

### Other PD Diagnostics

Many potential protein biomarkers in the blood and cerebrospinal fluid have been pursued for the diagnosis and staging of PD. DJ-1 and α-synuclein, two proteins critically involved in PD pathogenesis, have been tested as potential disease biomarkers, but results have been inconsistent [23], [24]. CSF levels of α-synuclein show a decrease or no change between patients with PD and controls [25]–[27]. Even α -synuclein-reactive antibodies have been pursued as diagnostic biomarkers of PD. Studies have shown significantly higher antibody levels towards monomeric α-synuclein in the sera of PD patients when compared to controls, but these responses decreased with PD progression [28]. Several other potential protein biomarkers for PD are currently being investigated but the results have been highly variable and somewhat non-specific. The detection of disease-specific serum autoantibodies with the potential to accurately and specifically diagnose PD presents a hitherto unexplored new avenue for continued research into PD etiology, diagnosis, and treatment.

### Conclusion

There is a profound need for accurate and specific biomarkers to aid in the primary diagnosis of PD. The 10 autoantibody biomarkers identified here have demonstrated a diagnostic sensitivity of 93.1% and specificity of 100% in differentiating PD sera from healthy controls. Similar accuracies were obtained when differentiating PD sera from other diseases. A reliable blood test for PD will have a tremendous clinical impact, not only to patients and their physicians, but also to pharmaceutical companies trying to gauge the effectiveness of disease-modifying drugs in clinical-trials. The relative non-invasiveness, broad availability, low cost, and versatility of protein microarrays make a technology of this kind well-suited for incorporation into routine health care. We hope that early, perhaps even pre-symptomatic, screening methods can be established for the betterment of patients. More than that, we view serum autoantibodies as an exciting new class of pathologically-relevant molecules that can be explored for better comprehension of disease mechanisms and potential therapies.
